# Bioinformatics analysis of calcium-dependent protein kinase 4 (CDPK4) as *Toxoplasma gondii* vaccine target

**DOI:** 10.1186/s13104-021-05467-1

**Published:** 2021-02-06

**Authors:** Masoud Foroutan, Ali Dalir Ghaffari, Shahrzad Soltani, Hamidreza Majidiani, Ali Taghipour, Mohamad Sabaghan

**Affiliations:** 1 USERN Office, Abadan Faculty of Medical Sciences, Abadan, Iran; 2 Department of Parasitology, Faculty of Medical Sciences, Tarbiat Modares University, P. O. Box 14115-111, Tehran, Iran; 3 Zoonotic Diseases Research Center, Ilam University of Medical Sciences, Ilam, Iran; 4 Behbahan Faculty of Medical Sciences, Behbahan, Iran

**Keywords:** *Toxoplasma gondii*, Calcium-dependent protein kinase 4, Bioinformatics analysis, In silico, Vaccine

## Abstract

**Objectives:**

*Toxoplasma gondii* (*T. gondii*), an obligate intracellular apicomplexan parasite, could affect numerous warm-blooded animals, such as humans. Calcium-dependent protein kinases (CDPKs) are essential Ca^2+^ signaling mediators and participate in parasite host cell egress, outer membrane motility, invasion, and cell division.

**Results:**

Several bioinformatics online servers were employed to analyze and predict the important properties of CDPK4 protein. The findings revealed that CDPK4 peptide has 1158 amino acid residues with average molecular weight (MW) of 126.331 KDa. The aliphatic index and GRAVY for this protein were estimated at 66.82 and – 0.650, respectively. The findings revealed that the CDPK4 protein comprised 30.14% and 34.97% alpha-helix, 59.84% and 53.54% random coils, and 10.02% and 11.49% extended strand with SOPMA and GOR4 tools, respectively. Ramachandran plot output showed 87.87%, 8.40%, and 3.73% of amino acid residues in the favored, allowed, and outlier regions, respectively. Also, several potential B and T-cell epitopes were predicted for CDPK4 protein through different bioinformatics tools. Also, antigenicity and allergenicity evaluation demonstrated that this protein has immunogenic and non-allergenic nature. This paper presents a basis for further studies, thereby provides a fundamental basis for the development of an effective vaccine against *T. gondii* infection.

## Introduction

*Toxoplasma gondii* (*T. gondii*), a compulsory intracellular parasite, could affect nearly all warm-blooded animals, such as humans [1, 2]. The current drugs against toxoplasmosis are not effective enough and are associated with serious side effects [3]. Therefore, the finding and development of an effective vaccine have high priority and is critically required to limit *T. gondii* infection [4–6]. Calcium-dependent protein kinases (CDPKs) are a category of serine/threonine kinases found only in plants and protists like ciliates and apicomplexan parasites [7, 8]. Multiple CDPKs (the most important household proteins) have been identified in apicomplexan protists, especially in *T. gondii*. The CDPKs are essential Ca^2+^ signaling mediators and participate in parasite host cell egress, outer membrane motility, invasion, and cell division [9–12]. The CDPK family was regarded as a good choice for anti-*Toxoplasma* medications and an appropriate option for the design of vaccines [13–17]. There is no report regarding immunization with CDPK4 in experimental animals yet. However, in several studies, vaccination with CDPK1 [13, 18, 19], CDPK2 [20], CDPK3 [15, 21, 22], CDPK5 [14], and CDPK6 [16] induced strong humoral and cellular responses and prolonged the survival time in mouse models.

Predicting epitopes is highly important for the determination of an antigen’s immunogenicity in vaccine development. So, bioinformatics tools and online resources can enable researchers to predict and recognize the potential epitopes of B- and T-cells [23–26]. Bioinformatics has recently become the preferred interdisciplinary new science for analyzing biological data using defined computer science, mathematics, statistics, physics, biology, medicine technologies and algorithms [23, 26]. Thus, the current study was performed to analyze the several important features of CDPK4 protein using different bioinformatics servers.

## Methodology

### Retrieval of CDPK4 protein sequence of *T. gondii*

First, the complete amino acid sequence of *T. gondii* CDPK4 protein was attained from ToxoDB server (https://toxodb.org/toxo/).

### Physicochemical evaluations

The physicochemical characteristics of the CDPK4 protein are of crucial significance in the evaluation of its aliphatic index, half-life, theoretical isoelectric point (pI), a grand average of hydropathicity (GRAVY), and electric charge distribution. The mentioned features were explored by ProtParam server [27, 28].

### Prediction of acylation and phosphorylation sites of CDPK4

In order to predict acylation and phosphorylation sites of CDPK4 protein, CSS-Palm and NetPhos online tools were employed, respectively [29, 30].

### Transmembrane domains and subcellular location prediction

The transmembrane domains of CDPK4 were examined by the TMHMM server v.2.0. Moreover, the PSORT II server was applied to predict the subcellular position of the CDPK4 protein.

### Prediction of secondary structure

Garnier–Osguthorpe–Robson 4 (GOR4) [31], SOPMA [32], and PSIPRED [33] online servers were utilized for prediction of the secondary structure of the CDPK4 protein.

### Construction of the 3D model

3D models play a decisive role in the development of vaccines. In this case, the SWISS-MODEL webserver was applied to build the three-dimensional models of the CDPK4 protein through a homology modeling approach [34].

### Refining and validating the 3D modeled structure

The proper SWISS-MODEL-constructed model was selected and modified by GalaxyRefine to attain high-quality template-based protein predictions [35]. The Ramachandran plot was utilized to validate the three-dimensional construct of the CDPK4 protein through the use of the SWISS-MODEL software [36]. The quality of the model was checked out using ProSAweb [37, 38].

### Prediction of B-cell epitopes

Various databases were employed for the analysis of the linear epitopes of the B-cells, including, Bcepred [39], ABCpred [40], SVMTriP [41], and the immune epitope database (IEDB). Furthermore, discontinuous B-cell epitopes were predicted by ElliPro [42].

### Prediction of major histocompatibility complex-I (MHC-I) and MHC-II epitopes

In this study, the IEDB [43] and NetMHCcons 1.1 [44] online services were used to predict the binding affinity of CDPK4 peptides toward the MHC class I. Furthermore, IEDB [45] and NetMHCIIpan 3.2 [46] servers were exploited to examine the 15-mer T- cell epitopes of H-2-IEd, H2IAd, and H2IAb mouse alleles.

### Detection of the CTL epitopes

To activate the immune system, an antigen should be first presented on the MHC-I surface. So, the choice of cytotoxic T lymphocyte (CTL) epitopes plays a decisive role in designing a vaccine. To this end, a free web server CTLpred [47] was utilized.

### Allergenicity, immunogenicity, and solubility evaluation

The possible allergenicity of the CDPK4 protein was explored utilizing AllerTOP [48] and AlgPred [28] servers. Protein antigenicity of CDPK4 protein was estimated using ANTIGENpro [49] and VaxiJen v. 2.0 [50] servers. Furthermore, we used the SOLpro online site to determine the protein solubility [38].

## Results and discussion

### Initial overview of the protein CDPK4

The amino acid sequence of CDPK4 protein was determined by the ToxoDB server under the accession ID: TGME49_237890. The CDPK4 protein includes 1158 amino acid residues with an estimated molecular weight of 126.331 KDa (antigens which have MW of < 5–10 KDa are considered as weak immunogens) [51], whereas its theoretical pI was 9.15. The total number of residues with the negative and positive charge was 145 and 167, respectively. Its half-life was predicted as 30 h, > 20 h, and > 10 h in mammalian reticulocytes cells (in vitro), yeast cells, and *E. coli*, respectively. Based on the instability index results (58.84), the CDPK4 protein is unstable. The relatively good estimated aliphatic index value of 66.82 indicates the thermostability of the protein. According to GRAVY data (− 0.650), CDPK4 protein exhibits hydrophilic features.

### Prediction of PTM sites of CDPK4

**Fig. 1:**
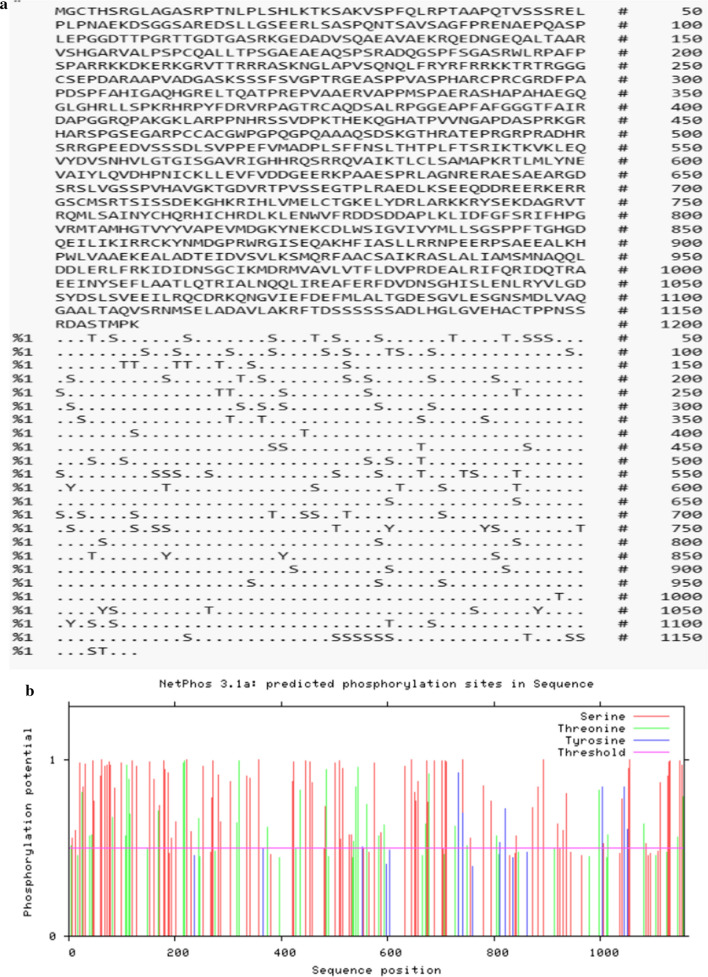
The output from the NePhos server for CDPK4 phosphorylation sites. **a** The number of predicted S/T/Y phosphorylation sites; Residues having a prediction score above the threshold is indicated by ‘S’, ‘T’ or ‘Y’, respectively. **b** Graphic showing the phosphorylation prediction sites

As it is evident, PTMs have important roles in cellular control processes [52]. Based on the findings, 137 phosphorylation and 21 acylation sites were predicted on CDPK4 sequence, suggesting that these sites may control several functions of the protein and affect protein activity (Fig. 1; Additional file 1: Table S1).

### Identification of transmembrane domains and subcellular location

The data obtained from TMHMM server v. 2.0 indicated no transmembrane domain in the CDPK4 sequence (Additional file 2: Figure S1). Furthermore, based on the PSORT II prediction, the subcellular location of CDPK4 is as follows: 82.6% nuclear, 8.7% plasma membrane, 4.3% mitochondrial, and 4.3% cytoskeletal.

### Secondary structure assessment

It should be noted that determining the protein secondary structure by introducing special constraints, such as beta-turn or alpha helix, is a key step in the assessment of the tertiary structure. The findings showed the CDPK4 protein comprised 30.14% (349/1158) and 34.97% (405/1158) alpha-helix, 59.84% (693/1158) and 53.54% (620/1158) random coils, and 10.02% (116/1158) and 11.49% (133/1158) extended strand by SOPMA and GOR4 servers, respectively (Additional file 2: Figures S2 and S3). The findings from the PSIPRED server are depicted in Additional file 2: Figure S4. It is apparent that alpha-helix and beta-turn placed in the protein’s internal portions, with high hydrogen bond-energy, will maintain the protein's structure resulting in a better interaction with antibodies [38, 53]. The principal biological behavior of the proteins is focused on their spatial structure. Knowledge of protein structures and awareness the relationships between structures and functions are important [38].

### 3D model analysis

Following the analysis, five 3D models were established for the CDPK4 sequence among which, the one with the highest identity was chosen. The chosen template exhibited a 34.99% sequence identity. The SWISS-MODEL results are presented in Additional file 2: Figure S5.

### Refinement and validation of the tertiary structure

**Fig. 2:**
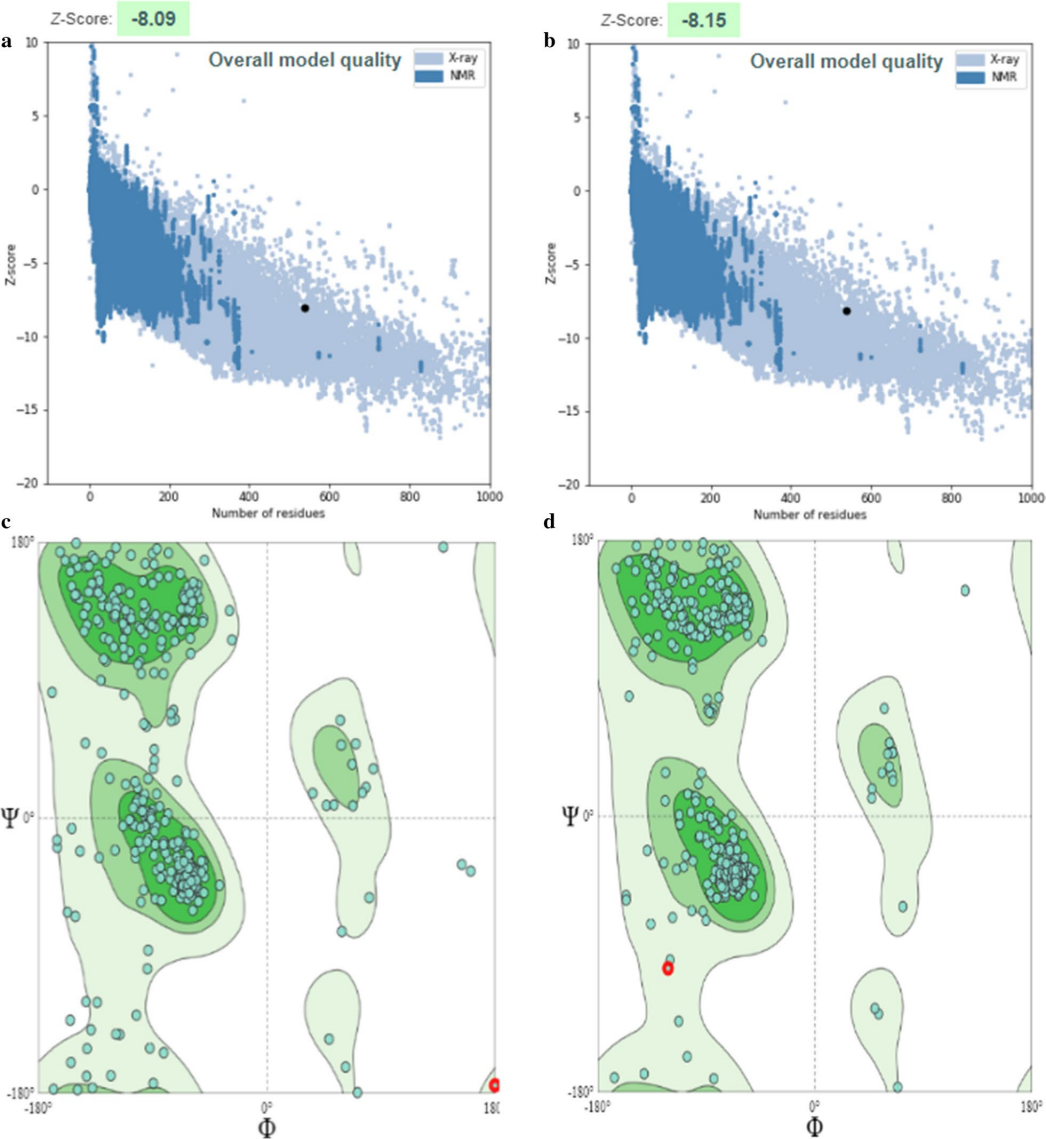
Validation of CDPK4 protein 3D structure using Ramachandran plot. **a** The Z-score plot for 3D structure of predicted protein before refinement with ProSA-web server. **b** The Z-score plot for 3D structure of predicted protein after refinement with ProSA-web server. **c** The analysis of Ramachandran plot using SWISS-MODEL server in initial model showed 87.87%, 8.40% and 3.73% of residues were located in favored, allowed and outlier regions, respectively. **d** The results after refinement were changed as follow: 95.34%, 3.54% and 1.12% of residues were located in favored, allowed and outlier regions, respectively

The GalaxyRefine software was employed to refine the tertiary structures. According to the results of the Ramachandran plot and ProSAweb servers, an enhancement was observed in the quality of the three-dimensional structure after the refinement. Prior to the refinement process, validation of the protein using the SWISS-MODEL tool showed that 87.87% of residues were situated in favored regions, while 8.40% and 3.73% of them lied in allowed and outlier regions, respectively, verifying its immunogenic efficiency (Fig. 2c). The post-refinement exploration of Ramachandran plots demonstrated that 95.34% of the residues lied within the favored regions, whereas 3.54% of them were in the allowed regions and only 1.12% of the residues were placed the outlier regions (Fig. 2d). The Z-score is indicative of the model quality; this parameter was − 8.09 in the initial model (based on the ProSA-web), and the majority of residues lied in the favored regions (Fig. 2a). Further improvement in the quality of the 3D structure after refinement can be also inferred from the Z-score value (− 8.15) (Fig. 2b).

### B-cell epitopes prediction

The epitope prediction could offer invaluable data that can be used to identify the immunogenic peptides. The Bcepred-determined linear B-cell epitopes are presented in Additional file 1: Table S2; while the results obtained from the ABCpred server are listed in Additional file 1: Table S3. The greater peptide score indicates the higher possibility of being an epitope. According to the IEDB online tool, the mean scores of hydrophilicity, antigenicity, beta-turn, bepipred linear epitope prediction, flexibility, and surface accessibility of the CDPK4 protein are 2.381, 1.016, 1.012, 0.350, 1.013, and 1.000, respectively (Additional file 2: Figure S6). The SVMTriP-derived results are also tabulated in Additional file 1: Table S4. The analysis of linear B-cell epitopes demonstrated that the CDPK4 protein contains favorable epitopes and appropriate indices. The Bcepred’s estimation accuracy of models based on various properties differs from 52.92% to 57.53%. This server also assists to forecast epitopes of B-cells using physicochemical features [38, 39]. Another valuable step for the in silico analysis is the identification of the conformational epitopes needed for antibody-antigen interaction [30]. In this case, the application of ElliPro tool resulted in five discontinuous B-cell epitopes (Table 1).

**Table 1:** Conformational B cell epitopes of CDPK4 protein predicted by ElliPro server

Residues	Number of Residues	Score	3D structure
A:K627, A:P628, A:A629, A:A630, A:E631, A:S632, A:P633, A:R634, A:L635, A:A636, A:G637, A:N638, A:R639, A:E640, A:R641, A:A642, A:E643, A:S644, A:A645, A:E646, A:A647, A:R648, A:G649, A:D650, A:S651, A:R652, A:S653, A:L654, A:V655, A:G656, A:S657, A:S658, A:P659, A:V660, A:H661, A:A662, A:V663, A:G664, A:K665, A:T666, A:G667, A:D668, A:V669, A:R670, A:T671, A:P672, A:V673, A:S674, A:S675, A:E676, A:G677, A:T678, A:P679, A:L680, A:R681, A:A682, A:E683, A:D684, A:L685, A:K686, A:S687, A:E688, A:D691, A:D692, A:R693, A:E694	66	0.809	
A:L950, A:D951, A:D952, A:L953, A:E954, A:R955, A:L956, A:F957, A:R958, A:K959, A:I960, A:D961, A:I962, A:D963, A:N964, A:S965, A:G966, A:C967, A:I968, A:K969, A:M970, A:D971, A:R972, A:M973, A:V974, A:A975, A:V976, A:F980, A:P984, A:R985, A:D986, A:A988, A:L989, A:R990, A:I991, A:F992, A:R994, A:I995, A:D996, A:Q997, A:T998, A:R999, A:A1000, A:E1001, A:E1002, A:I1003, A:N1004, A:Y1005, A:S1006, A:E1007, A:F1008, A:L1009, A:A1010, A:A1011, A:T1012, A:L1013, A:Q1014, A:T1015	58	0.773	
A:F845, A:G847, A:H848, A:G849, A:E852, A:I855, A:K856, A:R858, A:R859, A:D1032, A:V1033, A:D1034, A:N1035, A:S1036, A:G1037, A:H1038, A:I1039, A:S1040, A:L1041, A:E1042, A:N1043, A:L1044, A:R1045, A:G1049, A:Y1052, A:D1053, A:S1054, A:L1055, A:S1056, A:V1057, A:E1058, A:E1059, A:I1060, A:L1061, A:R1062, A:Q1063, A:C1064, A:D1065, A:K1067, A:Q1068, A:N1069, A:G1070, A:V1071, A:I1072, A:E1073, A:F1074, A:D1075, A:E1076, A:M1078, A:L1079, A:A1080, A:T1082, A:G1083, A:D1084	54	0.679	
A:R740, A:Y741, A:S742, A:E743, A:K744, A:D745, A:G747, A:R748, A:R751, A:N758, A:Q762, A:D779, A:D780, A:S781, A:D782, A:D783, A:A784, A:N823, A:E824, A:K825, A:L839, A:S840, A:P844, A:C860, A:Y862, A:N863, A:M864, A:D865, A:G866, A:P867, A:R868, A:W869, A:R870, A:G871, A:I872, A:S873, A:E874, A:Q875, A:A876, A:K877, A:H878, A:F879, A:I880, A:A881, A:S882, A:L884, A:R885, A:R886, A:N887, A:P888, A:E889, A:E890, A:R891, A:P892, A:S893, A:A894, A:E895, A:E896, A:A897, A:L898, A:K899, A:H900, A:P901, A:W902, A:L903, A:V904, A:A905, A:A906, A:E907, A:K908, A:E909, A:A910, A:L911, A:A912, A:D913, A:T914, A:E915, A:I916, A:D917, A:V918, A:S919, A:K922	82	0.667	
A:V551, A:Y552, A:H572, A:R573, A:Q574, A:S575, A:R576, A:R577	8	0.569	

### Analysis of MHC-I and MHC-II molecules

The connection of peptides to MHC molecules is an important step in the presentation of antigens to T-cells and also a key step in the choice of potential epitopes. Additional file 1: Tables S5 and S6, respectively indicate the lowest percentile ranks for CDPK4 MHC-I and MHC-II as obtained from the IEDB site. The results derived from NetMHCcons and NetMHCIIpan are also listed in Additional file 1: Tables S7 and S8, respectively. In general, the lower percentile ranks (or IC_50_ values) indicate the higher level affinity, which represents a better T-cell epitopes and vice versa [28]. Based on the bioinformatics analyses, CDPK4 T-cell epitopes could strongly bind to MHC-I and MHC-II molecules. Since *T. gondii* is considered as an intracellular protozoa, the cellular immunity mediated by the T-cells have a pivotal role against this microorganism [54]. It is therefore extremely essential for the development of an effective vaccine against *T. gondii* to explain which type of T-cell-mediated immune response is participated [38, 54].

### Prediction of the CTL epitopes

The CTLpred server was utilized to select 10 high-rank and suitable epitopes to analyze the CTL epitope. The details are mentioned in Additional file 1: Table S9.

### Allergenicity, immunogenicity, and solubility analysis

CDPK4 protein could exhibit high immunogenicity as its antigenicity score was determined 0.780 and 0.622 (through the use of ANTIGENpro and VaxiJen servers), respectively. AllerTOP and AlgPred servers suggest the non-allergic features of this protein. The ability to determine allergenicity is important to make sure that candidates for vaccines are low in allergenicity [38]. Based on the SOLpro output, the solubility of the CDPK4 protein was determined as 0.7087.

## Conclusion

This paper provided a detailed explanation of the fundamental aspects of CDPK4 protein, such as physicochemical characteristics, transmembrane domains, secondary and tertiary structures, B- and T-epitopes, and other features of CDPK4, using bioinformatics servers. Based on the findings, CDPK4 protein revealed an acceptable antigenicity score. Also, this protein contains many good epitopes of B- and T-cells, suggesting that CDPK4 can considered as an appropriate vaccine candidate against *T. gondii*. This research presented important fundamental and theoretical evidence for further in vivo investigations on the CDPK4 protein to establish an effective vaccine against acute and chronic *T. gondii* infection.

## Limitations

In this paper, only in silico analysis was performed. More studies are recommended for the development of an effective vaccine in vivo using the CDPK4 alone or combined with other antigens in the future. Also, a combination of immunodominant CDPK4 epitopes with various adjuvants and formulations will be useful.

## Supplementary Information

**Additional file 1: Table S1.** The acylation sites of CDPK4 sequence. **Table S2.** Epitopes predicted in CDPK4 protein by different parameters based on Bcepred online server. **Table S3.** The predicted B-cell epitopes via ABCpred tool. **Table S4.** Linear B-Cell epitope of the CDPK4 protein by SVMTriP. **Table S5.** IC_50_ values for CDPK4 binding to MHC class I molecules obtained using the IEDB. **Table S6.** IC_50_ values for CDPK4 binding to MHC class II molecules obtained using the IEDB. **Table S7.** Details of selected MHC-I T-cell epitope of *T. gondii* CDPK4 protein sequence using NetMHCcons server. **Table S8.** Details of selected MHC-II T-cell epitope of *T. gondii* CDPK4 protein sequence using NetMHCIIpan server. **Table S9.** Predicted CDPK4 epitopes by CTLpred.

**Additional file 2: Figure S1.** Transmembrane domains expected in CDPK4 protein. **(A)** Some statistics and a list of the location of the predicted transmembrane helices and the predicted location of the intervening loop regions. Length: the length of the protein sequence; Number of predicted TMHs: The number of predicted transmembrane helices; Exp number of AAs in TMHs: The expected number of amino acids in transmembrane helices. If this number is larger than 18 it is very likely to be a transmembrane protein (OR have a signal peptide); Exp number, first 60 AAs: The expected number of amino acids in transmembrane helices in the first 60 amino acids of the protein. If this number more than a few, you should be warned that a predicted transmembrane helix in the N-term could be a signal peptide; Total prob of N-in: The total probability that the N-term is on the cytoplasmic side of the membrane; **(B)** Analysis of the transmembrane domains of CDPK4. **Figure S2. (A)** The results of the GOR4 server suggested that CDPK4 contains 34.97% alpha helix (Hh), 11.49% extended strand (Ee) and 53.54% random coils (Cc) in secondary structure; **(B)** Graphical finding from prediction of secondary structure of CDPK4 using GOR4. **Figure S3. (A)** The results of the SOPMA server suggested that CDPK4 contains 30.14% alpha helix (Hh), 10.02% extended strand (Ee) and 59.84% random coils (Cc) in secondary structure; **(B)** Graphical finding from prediction of secondary structure of CDPK4 using SOPMA server. **Figure S4.** Graphical output from prediction of secondary structure of CDPK4 using PSIPRED tool. **Figure S5.** Predicted 3D model by the SWISS-MODEL server. **Figure S6.** Propensity scale plots of CDPK4 protein. **(A)** Surface accessibility; **(B)** Antigenicity; **(C)** Bepipred linear epitope prediction; **(D)** Beta-turn; **(E)** Flexibility; **(F)** Hydrophilicity. On the graphs, the Y-axes indicate the corresponding score for each residue (averaged in the specified window), while the X-axes indicate the residue positions in the sequence. The higher residue score could be interpreted as having a higher likelihood that the residue would be part of the epitope (yellow color on the graphs). Green color (under the threshold) shows the unfavorable regions that are related to the properties of interest.

## Data Availability

The datasets generated and/or analysed during the current study are available in the ToxoDB repository (https://toxodb.org/toxo/app/record/gene/TGME49_237890).

## Abbreviations

3D: Three-dimensional
CDPK: Calcium-dependent protein kinase
CTL: Cytotoxic T lymphocyte
GOR: Garnier–Osguthorpe–Robson
GRAVY: Grand average of hydropathicity
IC_50_: Half maximal inhibitory concentration
IEDB: Immune epitope database
MHC: Major histocompatibility complex
MW: Molecular weight
PDB: Protein data bank
pI: Isoelectric point
PTM: Post-translational modification
*T. gondii*: *Toxoplasma gondii*

## References

[1] Cenci-Goga BT, Rossitto PV, Sechi P, McCrindle CM, Cullor JS (2011). Toxoplasma in animals, food, and humans: an old parasite of new concern. Foodborne Pathog Dis.

[2] Foroutan M, Fakhri Y, Riahi SM, Ebrahimpour S, Namroodi S, Taghipour A, Spotin A, Gamble HR, Rostami A (2019). The global seroprevalence of *Toxoplasma gondii* in pigs: a systematic review and meta-analysis. Vet Parasitol.

[3] Antczak M, Dzitko K, Długońska H (2016). Human toxoplasmosis—searching for novel chemotherapeutics. Biomed Pharmacother.

[4] Zhang NZ, Wang M, Xu Y, Petersen E, Zhu XQ (2015). Recent advances in developing vaccines against *Toxoplasma gondii*: an update. Expert Rev Vaccines.

[5] Foroutan M, Ghaffarifar F, Sharifi Z, Dalimi A, Jorjani O (2019). Rhoptry antigens as *Toxoplasma gondii* vaccine target. Clin Exp Vaccine Res.

[6] Foroutan M, Zaki L, Ghaffarifar F (2018). Recent progress in microneme-based vaccines development against *Toxoplasma gondii*. Clin Exp Vaccine Res.

[7] Tzen M, Benarous R, Dupouy-Camet J, Roisin M (2007). A novel *Toxoplasma gondii* calcium-dependent protein kinase. Parasite.

[8] Billker O, Lourido S, Sibley LD (2009). Calcium-dependent signaling and kinases in apicomplexan parasites. Cell Host Microbe.

[9] Lourido S, Shuman J, Zhang C, Shokat KM, Hui R, Sibley LD (2010). Calcium-dependent protein kinase 1 is an essential regulator of exocytosis in Toxoplasma. Nature.

[10] Lourido S, Tang K, Sibley LD (2012). Distinct signalling pathways control *Toxoplasma egress* and host-cell invasion. EMBO J.

[11] Morlon-Guyot J, Berry L, Chen CT, Gubbels MJ, Lebrun M, Daher W (2014). The *T oxoplasma gondii* calcium-dependent protein kinase 7 is involved in early steps of parasite division and is crucial for parasite survival. Cell Microbiol.

[12] Uboldi AD, McCoy JM, Blume M, Gerlic M, Ferguson DJ, Dagley LF, Beahan CT, Stapleton DI, Gooley PR, Bacic A (2015). Regulation of starch stores by a Ca^2+^-dependent protein kinase is essential for viable cyst development in *Toxoplasma gondii*. Cell Host Microbe.

[13] Chen J, Li Z-Y, Huang S-Y, Petersen E, Song H-Q, Zhou D-H, Zhu X-Q (2014). Protective efficacy of *Toxoplasma gondiicalcium*-dependent protein kinase 1 (TgCDPK1) adjuvated with recombinant IL-15 and IL-21 against experimental toxoplasmosis in mice. BMC Infect Dis.

[14] Zhang N-Z, Huang S-Y, Xu Y, Chen J, Wang J-L, Tian W-P, Zhu X-Q (2014). Evaluation of immune responses in mice after DNA immunization with putative *Toxoplasma gondii* calcium-dependent protein kinase 5. Clin Vaccine Immunol.

[15] Zhang N-Z, Huang S-Y, Zhou D-H, Chen J, Xu Y, Tian W-P, Lu J, Zhu X-Q (2013). Protective immunity against *Toxoplasma gondii* induced by DNA immunization with the gene encoding a novel vaccine candidate: calcium-dependent protein kinase 3. BMC Infect Dis.

[16] Zhang N-Z, Xu Y, Wang M, Chen J, Huang S-Y, Gao Q, Zhu X-Q (2016). Vaccination with *Toxoplasma gondii* calcium-dependent protein kinase 6 and rhoptry protein 18 encapsulated in poly (lactide-*co*-glycolide) microspheres induces long-term protective immunity in mice. BMC Infect Dis.

[17] Foroutan M, Ghaffarifar F (2018). Calcium-dependent protein kinases are potential targets for *Toxoplasma gondii* vaccine. Clin Exp Vaccine Res.

[18] Chen J, Li ZY, Petersen E, Liu WG, Zhu XQ (2016). Co-administration of interleukins 7 and 15 with DNA vaccine improves protective immunity against *Toxoplasma gondii*. Exp Parasitol.

[19] Huang SY, Chen K, Wang JL, Yang B, Zhu XQ (2019). Evaluation of protective immunity induced by recombinant calcium-dependent protein kinase 1 (TgCDPK1) protein against acute toxoplasmosis in mice. Microb Pathog.

[20] Chen K, Wang JL, Huang SY, Yang WB, Zhu WN, Zhu XQ (2017). Immune responses and protection after DNA vaccination against *Toxoplasma gondii* calcium-dependent protein kinase 2 (TgCDPK2). Parasite.

[21] Zhang NZ, Gao Q, Wang M, Elsheikha HM, Wang B, Wang JL, Zhang FK, Hu LY, Zhu XQ (2018). Immunization with a DNA vaccine cocktail encoding TgPF, TgROP16, TgROP18, TgMIC6, and TgCDPK3 genes protects mice against chronic toxoplasmosis. Front Immunol.

[22] Wu M, An R, Chen Y, Chen T, Wen H, Yan Q, Shen J, Chen L, Du J (2019). Vaccination with recombinant *Toxoplasma gondii* CDPK3 induces protective immunity against experimental toxoplasmosis. Acta Trop.

[23] Flower DR, Macdonald IK, Ramakrishnan K, Davies MN, Doytchinova IA (2010). Computer aided selection of candidate vaccine antigens. Immun Res.

[24] Wang Y, Wang G, Cai J, Yin H (2016). Review on the identification and role of *Toxoplasma gondii* antigenic epitopes. Parasitol Res.

[25] Keyvani H, Ahmadi NA, Ranjbar MM, Ataei Kachooei S, Ghorban K, Dadmanesh M (2016). Immunoinformatics study of gp120 of human immunodeficiency virus type 1 subtype CRF35_AD isolated from Iranian patients. Arch Clin Infect Dis.

[26] Ranjbar MM, Nayeb Ali A, Ghorban K, Ghalyanchi Langeroudi A, Dadmanesh M, Amini H-R, Sedighi Moghaddam B (2015). Immnoinformatics: novel view in understanding of immune system function, databases and prediction of immunogenic epitopes. Koomesh.

[27] Gasteiger E, Hoogland C, Gattiker A, Wilkins MR, Appel RD, Bairoch A (2005). Protein identification and analysis tools on the ExPASy server. The proteomics protocols handbook.

[28] Majidiani H, Soltani S, Ghaffari AD, Sabaghan M, Taghipour A, Foroutan M (2020). In-depth computational analysis of calcium-dependent protein kinase 3 of *Toxoplasma gondii* provides promising targets for vaccination. Clin Exp Vaccine Res.

[29] Zhou J, Wang L, Zhou A, Lu G, Li Q, Wang Z, Zhu M, Zhou H, Cong H, He S (2016). Bioinformatics analysis and expression of a novel protein ROP48 in *Toxoplasma gondii*. Acta Parasitol.

[30] Foroutan M, Ghaffarifar F, Sharifi Z, Dalimi A, Pirestani M (2018). Bioinformatics analysis of ROP8 protein to improve vaccine design against *Toxoplasma gondii*. Infect Genet Evol J Mol Epidemiol Evol Genet Infect Dis.

[31] Garnier J, Gibrat J-F, Robson B (1996). [32] GOR method for predicting protein secondary structure from amino acid sequence. Methods in enzymology.

[32] Deléage G. ALIGNSEC: viewing protein secondary structure predictions within large multiple sequence alignments. Bioinformatics. 2017. 10.1093/bioinformatics/btx521 28961944

[33] McGuffin LJ, Bryson K, Jones DT (2000). The PSIPRED protein structure prediction server. Bioinformatics.

[34] Guex N, Peitsch MC, Schwede T (2009). Automated comparative protein structure modeling with SWISS-MODEL and Swiss-PdbViewer: a historical perspective. Electrophoresis.

[35] Park H, Seok C (2012). Refinement of unreliable local regions in template-based protein models. Proteins Struct Funct Bioinform.

[36] Bertoni M, Kiefer F, Biasini M, Bordoli L, Schwede T (2017). Modeling protein quaternary structure of homo-and hetero-oligomers beyond binary interactions by homology. Sci Rep.

[37] Wiederstein M, Sippl MJ (2007). ProSA-web: interactive web service for the recognition of errors in three-dimensional structures of proteins. Nucleic Acids Res.

[38] Ghaffari AD, Dalimi A, Ghaffarifar F, Pirestani M (2020). Structural predication and antigenic analysis of ROP16 protein utilizing immunoinformatics methods in order to identification of a vaccine against *Toxoplasma gondii*: An in silico approach. Microb Pathog.

[39] Saha S, Raghava GPS. BcePred: prediction of continuous B-cell epitopes in antigenic sequences using physico-chemical properties. In: International Conference on Artificial Immune Systems. Springer, Berlin, pp. 197–204; 2004.

[40] Saha S, Raghava GPS (2006). Prediction of continuous B-cell epitopes in an antigen using recurrent neural network. Proteins Struct Funct Bioinform.

[41] Yao B, Zhang L, Liang S, Zhang C (2012). SVMTriP: a method to predict antigenic epitopes using support vector machine to integrate tri-peptide similarity and propensity. PLoS ONE.

[42] Ponomarenko J, Bui H-H, Li W, Fusseder N, Bourne PE, Sette A, Peters B (2008). ElliPro: a new structure-based tool for the prediction of antibody epitopes. BMC Bioinform.

[43] Wang P, Sidney J, Kim Y, Sette A, Lund O, Nielsen M, Peters B (2010). Peptide binding predictions for HLA DR, DP and DQ molecules. BMC Bioinform.

[44] Karosiene E, Lundegaard C, Lund O, Nielsen M (2012). NetMHCcons: a consensus method for the major histocompatibility complex class I predictions. Immunogenetics.

[45] Wang P, Sidney J, Dow C, Mothé B, Sette A, Peters B (2008). A systematic assessment of MHC class II peptide binding predictions and evaluation of a consensus approach. PLoS Comput Biol.

[46] Jensen KK, Andreatta M, Marcatili P, Buus S, Greenbaum JA, Yan Z, Sette A, Peters B, Nielsen M (2018). Improved methods for predicting peptide binding affinity to MHC class II molecules. Immunology.

[47] Bhasin M, Raghava G (2004). Prediction of CTL epitopes using QM SVM and ANN techniques. Vaccine.

[48] Dimitrov I, Flower DR, Doytchinova I (2013). AllerTOP—a server for in silico prediction of allergens. BMC Bioinform BioMed Central.

[49] Magnan CN, Zeller M, Kayala MA, Vigil A, Randall A, Felgner PL, Baldi P (2010). High-throughput prediction of protein antigenicity using protein microarray data. Bioinformatics.

[50] Doytchinova IA, Flower DR (2007). VaxiJen: a server for prediction of protective antigens, tumour antigens and subunit vaccines. BMC Bioinform.

[51] Berzofsky JA. Immunogenicity and antigen structure. Fundamental immunology. 3rd ed. pp. 235–282; 1993.

[52] Lee T-Y, Hsu JB-K, Chang W-C, Wang T-Y, Hsu P-C, Huang H-D (2009). A comprehensive resource for integrating and displaying protein post-translational modifications. BMC Res Notes.

[53] Shaddel M, Ebrahimi M, Tabandeh MR (2018). Bioinformatics analysis of single and multi-hybrid epitopes of GRA-1, GRA-4, GRA-6 and GRA-7 proteins to improve DNA vaccine design against *Toxoplasma gondii*. J Parasit Dis.

[54] El-Kady IM (2011). T-cell immunity in human chronic toxoplasmosis. J Egypt Soc Parasitol.

