# Supplementary material for: Bioinformatics analysis of calcium-dependent protein kinase 4 (CDPK4) as Toxoplasma gondii vaccine target
Source: BMC Res Notes. 2021 Feb 6;14:50. doi: 10.1186/s13104-021-05467-1 (PMC7865105; doi:10.1186/s13104-021-05467-1)
Supplement: Supplementary file 1 — Additional file 1: Table S1. The acylation sites of CDPK4 sequence. Table S2. Epitopes predicted in CDPK4 protein by different parameters based on Bcepred online server. Table S3. The predicted B-cell epitopes via ABCpred tool. Table S4. Linear B-Cell epitope of the CDPK4 protein by SVMTriP. Table S5. IC50 values for CDPK4 binding to MHC class I molecules obtained using the IEDB. Table S6. IC50 values for CDPK4 binding to MHC class II molecules obtained using the IEDB. Table S7. Details of selected MHC-I T-cell epitope of T. gondii CDPK4 protein sequence using NetMHCcons server. Table S8. Details of selected MHC-II T-cell epitope of T. gondii CDPK4 protein sequence using NetMHCIIpan server. Table S9. Predicted CDPK4 epitopes by CTLpred. [file 13104_2021_5467_MOESM1_ESM.docx]

**Additional file 1: Table S1.** The acylation sites of CDPK4 sequence.

| **ID** | **Position** | **Peptide** | **Score** |
| --- | --- | --- | --- |
| TGME49_237890 CDPK4 [*T. gondii*] | 3 | *****MGCTHSRGLA | 18.572 |
| TGME49_237890 CDPK4 [*T. gondii*] | 163 | RVALPSPCQALLTPS | 0.561 |
| TGME49_237890 CDPK4 [*T. gondii*] | 251 | TRTRGGGCSEPDARA | 6.651 |
| TGME49_237890 CDPK4 [*T. gondii*] | 291 | VASPHARCPRCGRDF | 0.369 |
| TGME49_237890 CDPK4 [*T. gondii*] | 294 | PHARCPRCGRDFPAP | 9.624 |
| TGME49_237890 CDPK4 [*T. gondii*] | 376 | VRPAGTRCAQDSALR | 1.511 |
| TGME49_237890 CDPK4 [*T. gondii*] | 463 | GSEGARPCCACGWPG | 13.008 |
| TGME49_237890 CDPK4 [*T. gondii*] | 464 | SEGARPCCACGWPGP | 5.53 |
| TGME49_237890 CDPK4 [*T. gondii*] | 466 | GARPCCACGWPGPQG | 5.073 |
| TGME49_237890 CDPK4 [*T. gondii*] | 585 | QVAIKTLCLSAMAPK | 2.486 |
| TGME49_237890 CDPK4 [*T. gondii*] | 613 | QVDHPNICKLLEVFV | 1.721 |
| TGME49_237890 CDPK4 [*T. gondii*] | 703 | RKERRGSCMSRTSIS | 4.426 |
| TGME49_237890 CDPK4 [*T. gondii*] | 726 | IHLVMELCTGKELYD | 2.499 |
| TGME49_237890 CDPK4 [*T. gondii*] | 760 | MLSAINYCHQRHICH | 0.989 |
| TGME49_237890 CDPK4 [*T. gondii*] | 766 | YCHQRHICHRDLKLE | 0.137 |
| TGME49_237890 CDPK4 [*T. gondii*] | 826 | DGKYNEKCDLWSIGV | 7.011 |
| TGME49_237890 CDPK4 [*T. gondii*] | 860 | ILIKIRRCKYNMDGP | 0.862 |
| TGME49_237890 CDPK4 [*T. gondii*] | 930 | SMQRFAACSAIKRAS | 1.092 |
| TGME49_237890 CDPK4 [*T. gondii*] | 967 | IDIDNSGCIKMDRMV | 8.907 |
| TGME49_237890 CDPK4 [*T. gondii*] | 1064 | VEEILRQCDRKQNGV | 1.862 |
| TGME49_237890 CDPK4 [*T. gondii*] | 1144 | GLGVEHACTPPNSSR | 1.035 |

**Additional file 1: Table S2.** Epitopes predicted in CDPK4 protein by different parameters based on Bcepred online server.

| **Prediction parameter** | **Epitope sequence** |
| --- | --- |
| **Hydrophilicity** | GASRPTN; TVSSSRE; PNAEKDSGGSAREDS; SASPQNTSA; PRENAEPQAS; EPGGDTTPGRTTGDTGASRKGEDADVSQA; AEKRQEDNGEQA; TPSGAEAEAQSPSRADQGSP; PARRKKDKERKGR; TTRRRASKNG; RRKKTRTRGGGCSEPDARA; ADGASKSSS; GPTRGEASP; GAQHGRE; TQATPRE; SPAERAS; AHAEGQG; GTRCAQDSA; RDAPGGRQPAKGK; NHRSSVDPKTHEKQGHAT; NGAPDASPRKG; ARSPGSEGARP; QAAAQSDSKGTHRATEPRGRPRADHRSRRGPEEDVSSSD; HRQSRRQ; VDDGEERKPAAE; AGNRERAESAEARGDSRS; GKTGDVRT; EDLKSEEQDDREERKERRGSC; TSISSDEKGHKR; RKKRYSEKDAGRV; FRDDSDDAPL; DGKYNEKCD; TGHGDQE; RRNPEERPSAEEA; NAQQLDD; DIDNSGC; DVPRDEA; DQTRAEE; DVDNSGH; RQCDRKQNGV; LTGDESGV; ESGNSMD; FTDSSSSSSAD; TPPNSSRDAST. |
| **Flexibility** | SHLKTKSAK; AAPQTVSSSR; PLPNAEKDSGGSARE; LLGSEERLSASPQN; PGGDTTPGRTTGDTGASRKGE; EAVAEKRQEDNG; EAEAQSPSRADQ; AFPSPARRKKDKERKGRVTTRRRASK; FRYRFRRKKTRTRGGGC; PVADGASKSSSFSVGPTRGE; RLLSPKR; RDAPGGR; ARPPNHRSSVDPKTHE; GAPDASPRKGRHARSPGSEG; QAAAQSDSKGTHRATEPRGRPRADHRSRRGPEEDVSSSD; LFTSRIK; IGHHRQSRR; VFVDDGEER; PRLAGNRERAESAEARGDSR; GDVRTPVSSEG; EDLKSEEQDDREERKERRGSCMSRTSISSDEKGH; RLARKKRYSEKDA; WVFRDDS; SLLRRNPEER; EILRQCDRKQN; LALTGDESGVLESGNS; AQVSRNM; VLAKRFTDSSSSSS; HACTPPNSSRDA. |
| **Accessibility** | GASRPTNLP; SHLKTKSAKVSPFQLRPTAAPQTVSSSRELPLPNAEKDSGGSAREDS; GSEERLSASPQNTSA; FPRENAEPQASP; GDTTPGRTTGDTGASRKGEDAD; EAVAEKRQEDNGEQA; EAEAQSPSRADQGSP; FPSPARRKKDKERKGRVTTRRRASKNG; SQNQLFRYRFRRKKTRTRGG; SEPDARAA; PTRGEASPP; PHARCPR; RDFPAPD; QHGRELTQATPREPVA; PMSPAERASHAP; HRLLSPKRHRPYFDRVRP; QDSALRP; RDAPGGRQPAKGKLARPPNHRSSVDPKTHEKQGHATP; APDASPRKGRHARSPGSEG; PGPQGPQ; AAQSDSKGTHRATEPRGRPRADHRSRRGPEEDVSS; TSRIKTKVKLEQVYD; RIGHHRQSRRQVAIK; SAMAPKRT; VDDGEERKPAAESPRLAGNRERAESAEARGDSRSL; KTGDVRTPVSSE; TPLRAEDLKSEEQDDREERKERRGSC; SISSDEKGHKRIH; TGKELYDRLARKKRYSEKDAGRV; NYCHQRH; HRDLKLEN; FRDDSDDAPLK; MDGKYNEKCDL; TGHGDQE; IKIRRCKYNMDGPRWRGISEQAKHF; SLLRRNPEERPSAEEALKHP; EKEALAD; KSMQRFA; NAQQLDDLERLFRKIDIDN; LDVPRDEALR; FQRIDQTRAEEINYSE; NQQLIREAFERFDVDN; SLENLRY; LRQCDRKQNGV; TAQVSRNMSEL; AKRFTDSSS; TPPNSSRDASTMPK. |
| **Turns** | RPPNHRSS; YDVSNHVL; QVDHPNIC; RDDSDDAP; DIDNSGC; DVDNSGH; FTDSSSSSSA; CTPPNSSRD. |
| **Exposed surface** | LKTKSAKV; RENAEPQ; VAEKRQEDNGE; PSPARRKKDKERKGRVTTRRRASKN; RYRFRRKKTRTRG; LSPKRHRPYF; RQPAKGK; RPPNHRS; DPKTHEKQG; PDASPRKGRHAR; EPRGRPRADHRSRRGPEED; TSRIKTKVKLEQ; HHRQSRRQV; DDGEERKPAAE; NRERAES; EDLKSEEQDDREERKERRGS; SDEKGHKRI; YDRLARKKRYSEKDA; RDLKLEN; DGKYNEKCD; KIRRCKY; LRRNPEERPS; QRIDQTR; LRQCDRKQN. |
| **Polarity** | LSHLKTKSA; LLGSEERLSAS; FPRENAEPQ; GASRKGEDADV; EAVAEKRQEDNGE; RVSHGAR; PSPARRKKDKERKGRVTTRRRASKNG; LFRYRFRRKKTRTRGG; PHARCPRCGR; GAQHGRELTQ; REPVAAER; PAERASHAPAHAE; HRLLSPKRHRPYFDR; RQPAKGKLARPPNHRS; VDPKTHEKQGHAT; ASPRKGRHARSPG; DSKGTHRATEPRGRPRADHRSRRGPEEDV; TSRIKTKVKLEQV; AVRIGHHRQSRRQVAIK; FVDDGEERKPAAES; RLAGNRERAESAEARGDSR; LRAEDLKSEEQDDREERKERRGSCM; SSDEKGHKRIHLVMELCTGKELYDRLARKKRYSEKDAGRV; NYCHQRHICHRDLKLEN; VFRDDSDDAP; DGKYNEKCD; ILIKIRRCKYNM; RWRGISEQAKHFI; LLRRNPEERPSAEEALKHPW; EKEALAD; QLDDLERLFRKIDIDN; LDVPRDEALRI; QRIDQTRAEEINYSE; LIREAFERFDVDN; SVEEILRQCDRKQN; HGLGVEH. |
| **Antigenic propensity** | NLPLSHLK; KVSPFQLR; LGHRLLS; SDLSVPPEFVM; KVKLEQVYDVSNHVLG; IKTLCLS; IYLQVDHPNICKLLEVFVDD; SLVGSSPVH; VRTPVSS; HKRIHLVMELCT; YCHQRHICHRDLKL; PLKLIDF; HGTVYYV; LWSIGVIVYMLLSG; LIKIRRC; LKHPWLV; EIDVSVLKSM; VLVTFLDVP; LRYVLGDSYDSLSVEEIL; |

**Additional file 1: Table S3.** The predicted B-cell epitopes via ABCpred tool.

| Rank | Sequence | Start position | Score |
| --- | --- | --- | --- |
| 1 | KSSSFSVGPTRGEASPPVAS | 267 | 0.93 |
| 2 | VMDGKYNEKCDLWSIGVIVY | 817 | 0.92 |
| 2 | KLLEVFVDDGEERKPAAESP | 614 | 0.92 |
| 2 | PPEFVMADPLSFFNSLTHTP | 517 | 0.92 |
| 2 | RHRPYFDRVRPAGTRCAQDS | 361 | 0.92 |
| 3 | IKTLCLSAMAPKRTLMLYNE | 581 | 0.91 |
| 3 | ATPVVNGAPDASPRKGRHAR | 434 | 0.91 |
| 3 | APAHAEGQGLGHRLLSPKRH | 343 | 0.91 |
| 3 | GDTGASRKGEDADVSQAEAV | 114 | 0.91 |
| 4 | WVFRDDSDDAPLKLIDFGFS | 775 | 0.90 |
| 4 | SPHARCPRCGRDFPAPDSPF | 286 | 0.90 |
| 4 | FRYRFRRKKTRTRGGGCSEP | 235 | 0.90 |
| 5 | YDVSNHVLGTGISGAVRIGH | 552 | 0.89 |
| 5 | TEPRGRPRADHRSRRGPEED | 489 | 0.89 |
| 5 | GPQGPQAAAQSDSKGTHRAT | 470 | 0.89 |
| 5 | KERKGRVTTRRRASKNGLAP | 209 | 0.89 |
| 6 | YNMDGPRWRGISEQAKHFIA | 862 | 0.88 |
| 6 | ISGAVRIGHHRQSRRQVAIK | 563 | 0.88 |
| 6 | TAARVSHGARVALPSPCQAL | 147 | 0.88 |
| 6 | QVSRNMSELADAVLAKRFTD | 1107 | 0.88 |
| 7 | RMVAVLVTFLDVPRDEALRI | 972 | 0.87 |
| 7 | EIDVSVLKSMQRFAACSAIK | 915 | 0.87 |
| 7 | DVDNSGHISLENLRYVLGDS | 1032 | 0.87 |
| 8 | SMQRFAACSAIKRASLALIA | 923 | 0.86 |
| 8 | RNPEERPSAEEALKHPWLVA | 886 | 0.86 |
| 8 | GHHRQSRRQVAIKTLCLSAM | 570 | 0.86 |
| 8 | ARVALPSPCQALLTPSGAEA | 155 | 0.86 |
| 8 | LRQCDRKQNGVIEFDEFMLA | 1061 | 0.86 |
| 9 | RGSCMSRTSISSDEKGHKRI | 700 | 0.85 |
| 9 | ARSPGSEGARPCCACGWPGP | 452 | 0.85 |
| 9 | PTAAPQTVSSSRELPLPNAE | 37 | 0.85 |
| 9 | SPAERASHAPAHAEGQGLGH | 335 | 0.85 |
| 9 | AERVAPPMSPAERASHAPAH | 327 | 0.85 |
| 9 | QNGVIEFDEFMLALTGDESG | 1068 | 0.85 |
| 10 | LSASPQNTSAVSAGFPRENA | 75 | 0.84 |
| 10 | HRSRRGPEEDVSSSDLSVPP | 499 | 0.84 |
| 10 | AHIGAQHGRELTQATPREPV | 306 | 0.84 |
| 10 | GGGCSEPDARAAPVADGASK | 248 | 0.84 |
| 10 | VLGDSYDSLSVEEILRQCDR | 1047 | 0.84 |
| 11 | KVKLEQVYDVSNHVLGTGIS | 545 | 0.83 |
| 11 | PAKGKLARPPNHRSSVDPKT | 408 | 0.83 |
| 11 | RRASKNGLAPVSQNQLFRYR | 219 | 0.83 |
| 12 | TFLDVPRDEALRIFQRIDQT | 979 | 0.82 |
| 12 | YMLLSGSPPFTGHGDQEILI | 836 | 0.82 |
| 12 | KCDLWSIGVIVYMLLSGSPP | 825 | 0.82 |
| 12 | EARGDSRSLVGSSPVHAVGK | 646 | 0.82 |
| 13 | EQAKHFIASLLRRNPEERPS | 874 | 0.81 |
| 13 | GDQEILIKIRRCKYNMDGPR | 849 | 0.81 |
| 13 | DPLSFFNSLTHTPLFTSRIK | 524 | 0.81 |
| 13 | VRPAGTRCAQDSALRPGGEA | 369 | 0.81 |
| 13 | DVSQAEAVAEKRQEDNGEQA | 126 | 0.81 |
| 13 | KRFTDSSSSSSADLHGLGVE | 1122 | 0.81 |
| 13 | LALTGDESGVLESGNSMDLV | 1079 | 0.81 |
| 14 | KRASLALIAMSMNAQQLDDL | 934 | 0.80 |
| 14 | SLTHTPLFTSRIKTKVKLEQ | 531 | 0.80 |
| 14 | EEDVSSSDLSVPPEFVMADP | 506 | 0.80 |
| 14 | TPSGAEAEAQSPSRADQGSP | 168 | 0.80 |
| 15 | VDHPNICKLLEVFVDDGEER | 607 | 0.79 |
| 15 | AFPSPARRKKDKERKGRVTT | 198 | 0.79 |
| 15 | SPFSGASRWLRPAFPSPARR | 186 | 0.79 |
| 16 | RYSEKDAGRVTRQMLSAINY | 740 | 0.78 |
| 16 | EINYSEFLAATLQTRIALNQ | 1002 | 0.78 |
| 17 | GTVYYVAPEVMDGKYNEKCD | 808 | 0.77 |
| 17 | GHKRIHLVMELCTGKELYDR | 715 | 0.77 |
| 17 | PVHAVGKTGDVRTPVSSEGT | 659 | 0.77 |
| 17 | RKPAAESPRLAGNRERAESA | 626 | 0.77 |
| 17 | RPPNHRSSVDPKTHEKQGHA | 415 | 0.77 |
| 17 | RAAPVADGASKSSSFSVGPT | 257 | 0.77 |
| 17 | QQLIREAFERFDVDNSGHIS | 1021 | 0.77 |
| 17 | AATLQTRIALNQQLIREAFE | 1010 | 0.77 |
| 18 | DEALRIFQRIDQTRAEEINY | 986 | 0.76 |
| 18 | DLERLFRKIDIDNSGCIKMD | 952 | 0.76 |
| 18 | DDAPLKLIDFGFSRIFHPGV | 782 | 0.76 |
| 18 | RLAGNRERAESAEARGDSRS | 634 | 0.76 |
| 18 | DAPGGRQPAKGKLARPPNHR | 401 | 0.76 |
| 18 | SALRPGGEAPFAFGGGTFAI | 380 | 0.76 |
| 19 | ELPLPNAEKDSGGSAREDSL | 49 | 0.75 |
| 19 | APDASPRKGRHARSPGSEGA | 441 | 0.75 |

**Additional file 1: Table S4.** Linear B-Cell epitope of the CDPK4 protein by SVMTriP.

| Rank | Location | Epitope | Score | Recommend* |
| --- | --- | --- | --- | --- |
| 1 | 729 - 748 | KELYDRLARKKRYSEKDAGR | 1.000 | 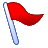 |
| 2 | 931 - 950 | SAIKRASLALIAMSMNAQQL | 0.874 | 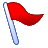 |
| 3 | 801 - 820 | VRMTAMHGTVYYVAPEVMDG | 0.831 | 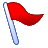 |
| 4 | 1105 - 1124 | TAQVSRNMSELADAVLAKRF | 0.816 | 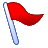 |
| 5 | 223 - 242 | KNGLAPVSQNQLFRYRFRRK | 0.675 |  |
| 6 | 1039 - 1058 | ISLENLRYVLGDSYDSLSVE | 0.598 |  |
| 7 | 572 - 591 | HRQSRRQVAIKTLCLSAMAP | 0.493 |  |
| 8 | 692 - 711 | DREERKERRGSCMSRTSISS | 0.452 |  |
| 9 | 136 - 155 | KRQEDNGEQALTAARVSHGA | 0.447 |  |
| 10 | 638 - 657 | NRERAESAEARGDSRSLVGS | 0.401 |  |

* The epitopes recommended are labeled by the flags

**Additional file 1: Table S5.** IC_50_ values for CDPK4 binding to MHC class I molecules obtained using the IEDB ^a^.

| **MHC II allele ^b^** | **Start-Stop ^c^** | **Peptide sequence** | **Percentile rank ^d^** |
| --- | --- | --- | --- |
|  | **CDPK4** |  | **CDPK4** |
| **H2-Db** | 1039-1048 | ISLENLRYVL | 0.13 |
|  | 76-85 | SASPQNTSAV | 0.47 |
|  | 77-86 | ASPQNTSAVS | 0.65 |
| **H2-Dd** | 273-282 | VGPTRGEASP | 0.59 |
|  | 60-69 | GGSAREDSLL | 1.18 |
|  | 357-366 | LSPKRHRPYF | 1.27 |
| **H2-Kb** | 1072-1081 | IEFDEFMLAL | 1.55 |
|  | 931-940 | SAIKRASLAL | 2.35 |
|  | 1003-1012 | INYSEFLAAT | 2.63 |
| **H2-Kd** | 1051-1060 | SYDSLSVEEI | 0.9 |
|  | 21-30 | SHLKTKSAKV | 0.96 |
|  | 835-844 | VYMLLSGSPP | 1.95 |
| **H2-Kk** | 907-916 | EKEALADTEI | 1.05 |
|  | 895-904 | EEALKHPWLV | 1.5 |
|  | 252-261 | SEPDARAAPV | 2.7 |
| **H2-Ld** | 383-392 | RPGGEAPFAF | 0.65 |
|  | 784-793 | APLKLIDFGF | 3.7 |
|  | 14-23 | APLKLIDFGF | 4.3 |

^a^ The immune epitope database (<http://tools.iedb.org/mhci/>).

^b^ H2-Db, H2-Dd, H2-Kb, H2-Kd, H2-Kk, and H2-Ld alleles are mouse MHC class I molecules.

^c^ Ten amino acids for analysis was used each time.

^d^ Low percentile rank = high level binding; high percentile rank = low level binding; IC_50_ values = percentile rank.

**Additional file 1: Table S6.** IC_50_ values for CDPK4 binding to MHC class II molecules obtained using the IEDB ^a^.

| **MHC II allele ^b^** | **Start-Stop ^c^** | **Peptide sequence** | **Percentile rank ^d^** |
| --- | --- | --- | --- |
|  | **CDPK4** |  | **CDPK4** |
| **H2-IAb** | 191-205 | ASRWLRPAFPSPARR | 0.56 |
|  | 192-206 | SRWLRPAFPSPARRK | 0.60 |
|  | 275-289 | PTRGEASPPVASPHA | 0.69 |
| **H2-IAd** | 931-945 | SAIKRASLALIAMSM | 0.40 |
|  | 932-946 | AIKRASLALIAMSMN | 0.42 |
|  | 930-944 | CSAIKRASLALIAMS | 0.58 |
| **H2-IEd** | 234-248 | LFRYRFRRKKTRTRG | 0.01 |
|  | 232-246 | NQLFRYRFRRKKTRT | 0.01 |
|  | 233-247 | QLFRYRFRRKKTRTR | 0.01 |

^a^ The immune epitope database (<http://tools.immuneepitope.org/mhcii>).

^b^ H2-IAb, H2-IAd, and H2-IEd alleles are mouse MHC class II molecules.

^c^ Fifteen amino acids for analysis was used each time.

^d^ Low percentile rank = high level binding; high percentile rank = low level binding; IC_50_ values = percentile rank.

**Additional file 1: Table S7.** Details of selected MHC-I T-cell epitope of *T. gondii* CDPK4 protein sequence using NetMHCcons server.

| Allele | Peptide sequence | IC_50_ (nM) | | % rank | Binding level ^a^ |
| --- | --- | --- | --- | --- | --- |
| H-2-Kb | INYSEFLAAT VSVLKSMQRF  ISLENLRYVL | | 392.49  430.30  484.69 | 1.50  2.00  2.00 | WB  WB  WB |
| H-2-Db | ISLENLRYVL IAMSMNAQQL  RASKNGLAPV | | 114.33  735.14  893.21 | 0.15  0.50  0.80 | SB  SB  WB |
| H-2-Kd | SYDSLSVEEI IYLQVDHPNI  NYSEFLAATL | | 132.31  277.63  401.08 | 0.40  0.40  0.40 | SB  SB  SB |
| H-2-Kk | DEALRIFQRI EEALKHPWLV  HEKQGHATPV | | 41.12  55.68  149.03 | 0.30  0.40  0.80 | SB  SB  WB |
| H-2-Dd | LSPKRHRPYF LAPVSQNQLF  MAPKRTLMLY | | 1414.68  3219.45  4883.05 | 0.05  0.25  0.50 | SB  SB  SB |
| H-2-Ld | APFAFGGGTF RPGGEAPFAF  RPTNLPLSHL | | 214.14  218.82  333.69 | 0.17  0.17  0.25 | SB  SB  SB |

^a^ SB: strong binders; WB: weak binders

**Additional file 1: Table S8.** Details of selected MHC-II T-cell epitope of *T. gondii* CDPK4 protein sequence using NetMHCIIpan server.

| Allele | Peptide sequence | IC_50_ (nM) | % rank | | Binding level ^a^ |
| --- | --- | --- | --- | --- | --- |
| H2-IAd | SAIKRASLALIAMSM  AIKRASLALIAMSMN  IKRASLALIAMSMNA  CSAIKRASLALIAMS  EQALTAARVSHGARV  KRASLALIAMSMNAQ  QALTAARVSHGARVA  GEQALTAARVSHGAR  ACSAIKRASLALIAM  NGEQALTAARVSHGA | 46.70  50.14  55.63  56.88  62.97  66.08  68.87  70.11  75.72  84.35 | | 0.06  0.07  0.12  0.12  0.17  0.20  0.25  0.25  0.30  0.40 | SB  SB  SB  SB  SB  SB  SB  SB  SB  SB |
| H2-IAb | SRWLRPAFPSPARRK ASRWLRPAFPSPARR  RWLRPAFPSPARRKK  GASRWLRPAFPSPAR  SGASRWLRPAFPSPA  WLRPAFPSPARRKKD  SRSLVGSSPVHAVGK  DSRSLVGSSPVHAVG  GDSRSLVGSSPVHAV  RSLVGSSPVHAVGKT | 110.99  113.98  132.48  135.12  192.56  198.33  236.09  253.38  259.83  300.30 | | 0.12  0.15  0.20  0.20  0.50  0.50  0.70  0.70  0.80  0.10 | SB  SB  SB  SB  SB  SB  SB  SB  SB  SB |
| H2-IEd | QLFRYRFRRKKTRTR  LFRYRFRRKKTRTRG  NQLFRYRFRRKKTRT  QNQLFRYRFRRKKTR  FRYRFRRKKTRTRGG  SQNQLFRYRFRRKKT  RYRFRRKKTRTRGGG  VSQNQLFRYRFRRKK  MQRFAACSAIKRASL  QAKHFIASLLRRNPE | 218.39  240.35  302.96  322.47  353.14  436.47  557.44  573.70  767.08  785.76 | | 0.04  0.05  0.10  0.12  0.15  0.30  0.50  0.50  1.00  1.00 | SB  SB  SB  SB  SB  SB  SB  SB  SB  SB |

^a^ SB: strong binders; WB: weak binders

**Additional file 1: Table S9.** Predicted CDPK4 epitopes by CTLpred ^a^.

| **Peptide rank** | **Start position ^b^** | **Sequence** | **Score (ANN/SVM) ^c^** |
| --- | --- | --- | --- |
| **1** | 597 | LYNEVAIYL | 0.81/1.8346356 |
| **2** | 1011 | ATLQTRIAL | 0.98/1.2002566 |
| **3** | 422 | SVDPKTHEK | 0.94/1.1599681 |
| **4** | 910 | ALADTEIDV | 0.75/1.1348842 |
| **5** | 1073 | EFDEFMLAL | 0.89/0.97820823 |
| **6** | 589 | MAPKRTLML | 0.98/0.88587145 |
| **7** | 198 | AFPSPARRK | 0.94/0.88161504 |
| **8** | 730 | ELYDRLARK | 0.80/1.0005003 |
| **9** | 805 | AMHGTVYYV | 0.41/1.3312945 |
| **10** | 347 | AEGQGLGHR | 0.96/0.74201453 |

^a^ CTLpred, available online at <http://www.imtech.res.in/raghava/ctlpred/index.html>.

^b^ Nine amino acids for analysis was used.

^c^ The default artificial neural network (ANN) and support vector machine (SVM) cut-off scores were set 0.51 and 0.36, respectively.
