# Supplementary material for: Bioinformatics analysis of calcium-dependent protein kinase 4 (CDPK4) as Toxoplasma gondii vaccine target
Source: BMC Res Notes. 2021 Feb 6;14:50. doi: 10.1186/s13104-021-05467-1 (PMC7865105; doi:10.1186/s13104-021-05467-1)
Supplement: Supplementary file 2 — Additional file 2: Figure S1. Transmembrane domains expected in CDPK4 protein. (A) Some statistics and a list of the location of the predicted transmembrane helices and the predicted location of the intervening loop regions. Length: the length of the protein sequence; Number of predicted TMHs: The number of predicted transmembrane helices; Exp number of AAs in TMHs: The expected number of amino acids in transmembrane helices. If this number is larger than 18 it is very likely to be a transmembrane protein (OR have a signal peptide); Exp number, first 60 AAs: The expected number of amino acids in transmembrane helices in the first 60 amino acids of the protein. If this number more than a few, you should be warned that a predicted transmembrane helix in the N-term could be a signal peptide; Total prob of N-in: The total probability that the N-term is on the cytoplasmic side of the membrane; (B) Analysis of the transmembrane domains of CDPK4. Figure S2. (A) The results of the GOR4 server suggested that CDPK4 contains 34.97% alpha helix (Hh), 11.49% extended strand (Ee) and 53.54% random coils (Cc) in secondary structure; (B) Graphical finding from prediction of secondary structure of CDPK4 using GOR4. Figure S3. (A) The results of the SOPMA server suggested that CDPK4 contains 30.14% alpha helix (Hh), 10.02% extended strand (Ee) and 59.84% random coils (Cc) in secondary structure; (B) Graphical finding from prediction of secondary structure of CDPK4 using SOPMA server. Figure S4. Graphical output from prediction of secondary structure of CDPK4 using PSIPRED tool. Figure S5. Predicted 3D model by the SWISS-MODEL server. Figure S6. Propensity scale plots of CDPK4 protein. (A) Surface accessibility; (B) Antigenicity; (C) Bepipred linear epitope prediction; (D) Beta-turn; (E) Flexibility; (F) Hydrophilicity. On the graphs, the Y-axes indicate the corresponding score for each residue (averaged in the specified window), while the X-axes indicate the residue positions in the [file 13104_2021_5467_MOESM2_ESM.docx]

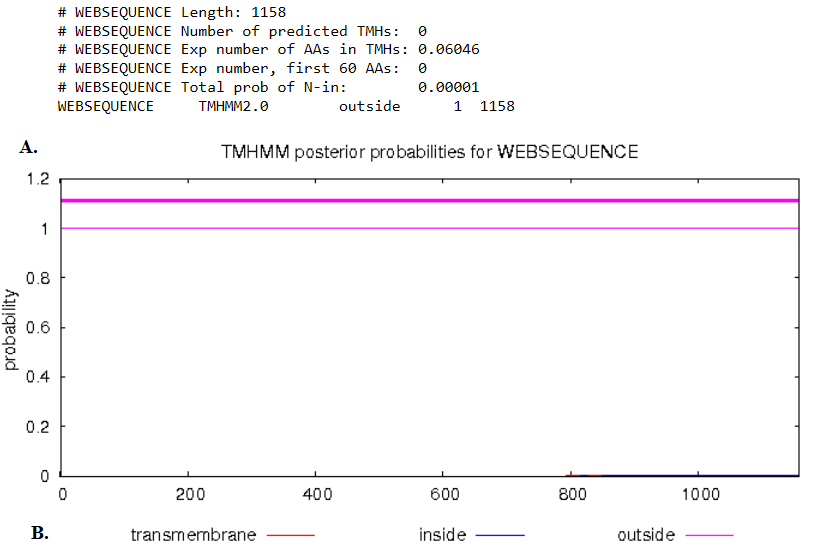


**Additional file 2: Figure S1.** **Transmembrane domains expected in CDPK4 protein.**

**(A)** Some statistics and a list of the location of the predicted transmembrane helices and the predicted location of the intervening loop regions. Length: the length of the protein sequence; Number of predicted TMHs: The number of predicted transmembrane helices; Exp number of AAs in TMHs: The expected number of amino acids in transmembrane helices. If this number is larger than 18 it is very likely to be a transmembrane protein (OR have a signal peptide); Exp number, first 60 AAs: The expected number of amino acids in transmembrane helices in the first 60 amino acids of the protein. If this number more than a few, you should be warned that a predicted transmembrane helix in the N-term could be a signal peptide; Total prob of N-in: The total probability that the N-term is on the cytoplasmic side of the membrane; **(B)** Analysis of the transmembrane domains of CDPK4.


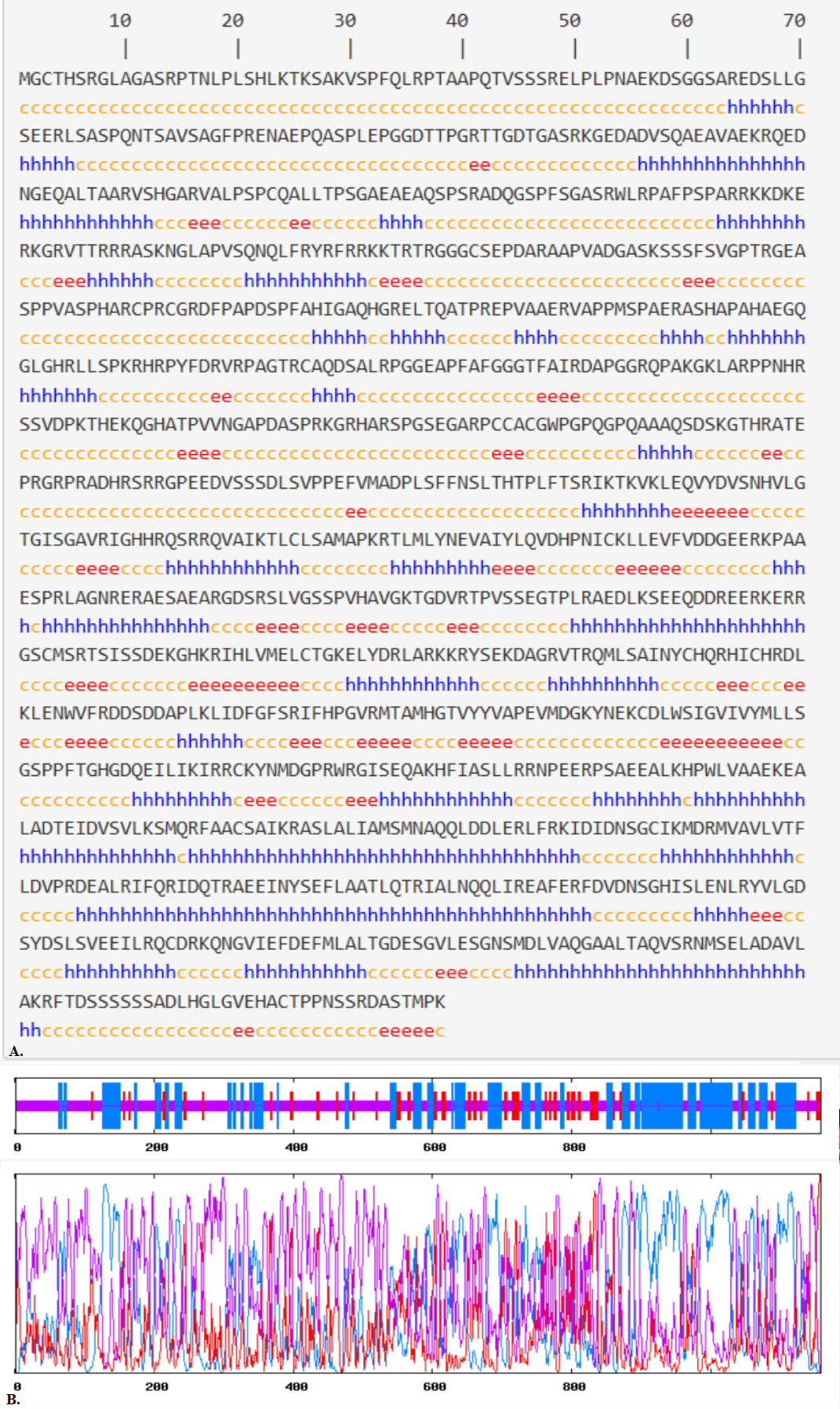


**Additional file 2: Figure S2.** **(A)** The results of the GOR4 server suggested that CDPK4 contains 34.97% alpha helix (Hh), 11.49% extended strand (Ee) and 53.54% random coils (Cc) in secondary structure; **(B)** Graphical finding from prediction of secondary structure of CDPK4 using GOR4.


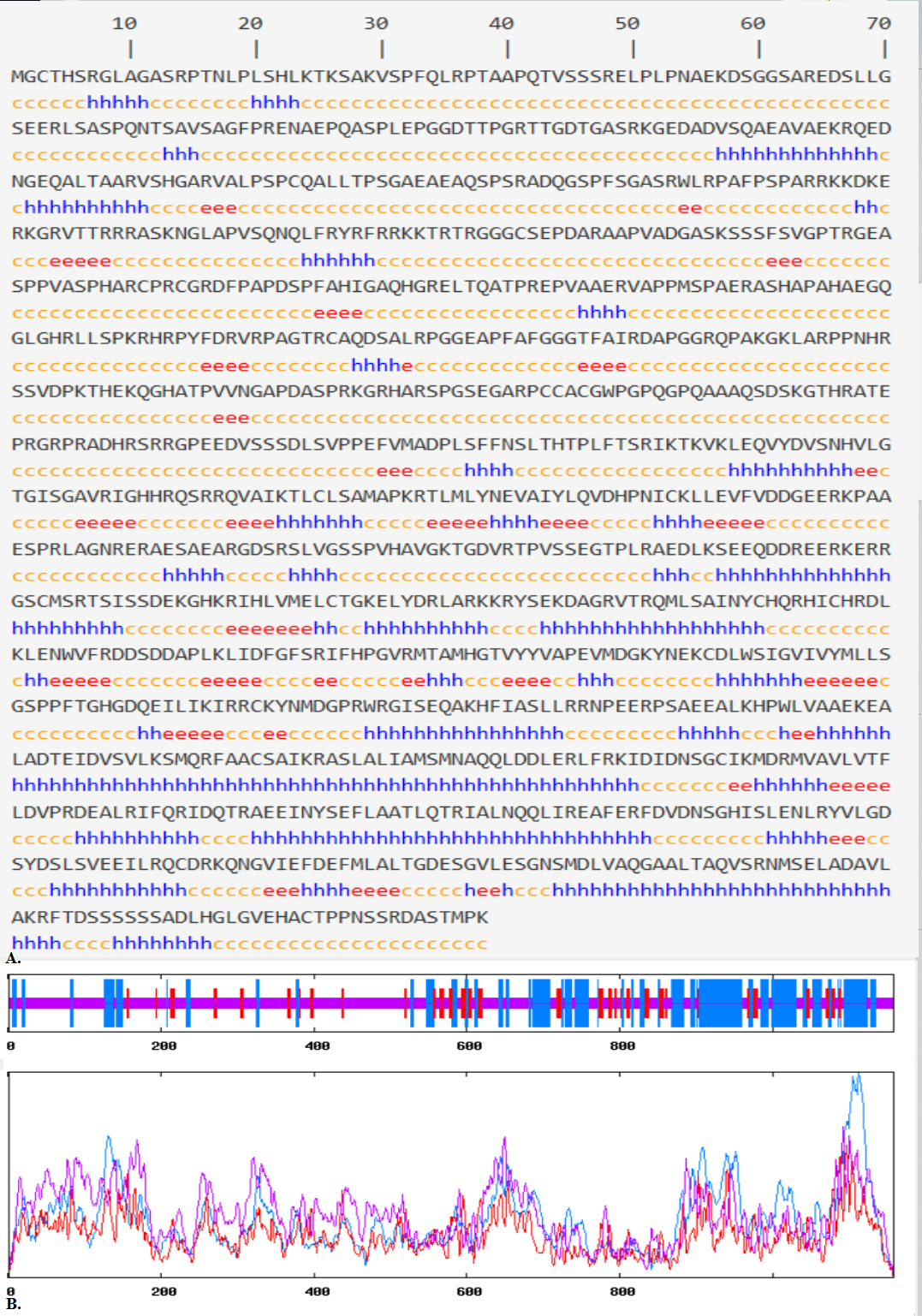


**Additional file 2: Figure S3. (A)** The results of the SOPMA server suggested that CDPK4 contains 30.14% alpha helix (Hh), 10.02% extended strand (Ee) and 59.84% random coils (Cc) in secondary structure; **(B)** Graphical finding from prediction of secondary structure of CDPK4 using SOPMA server.


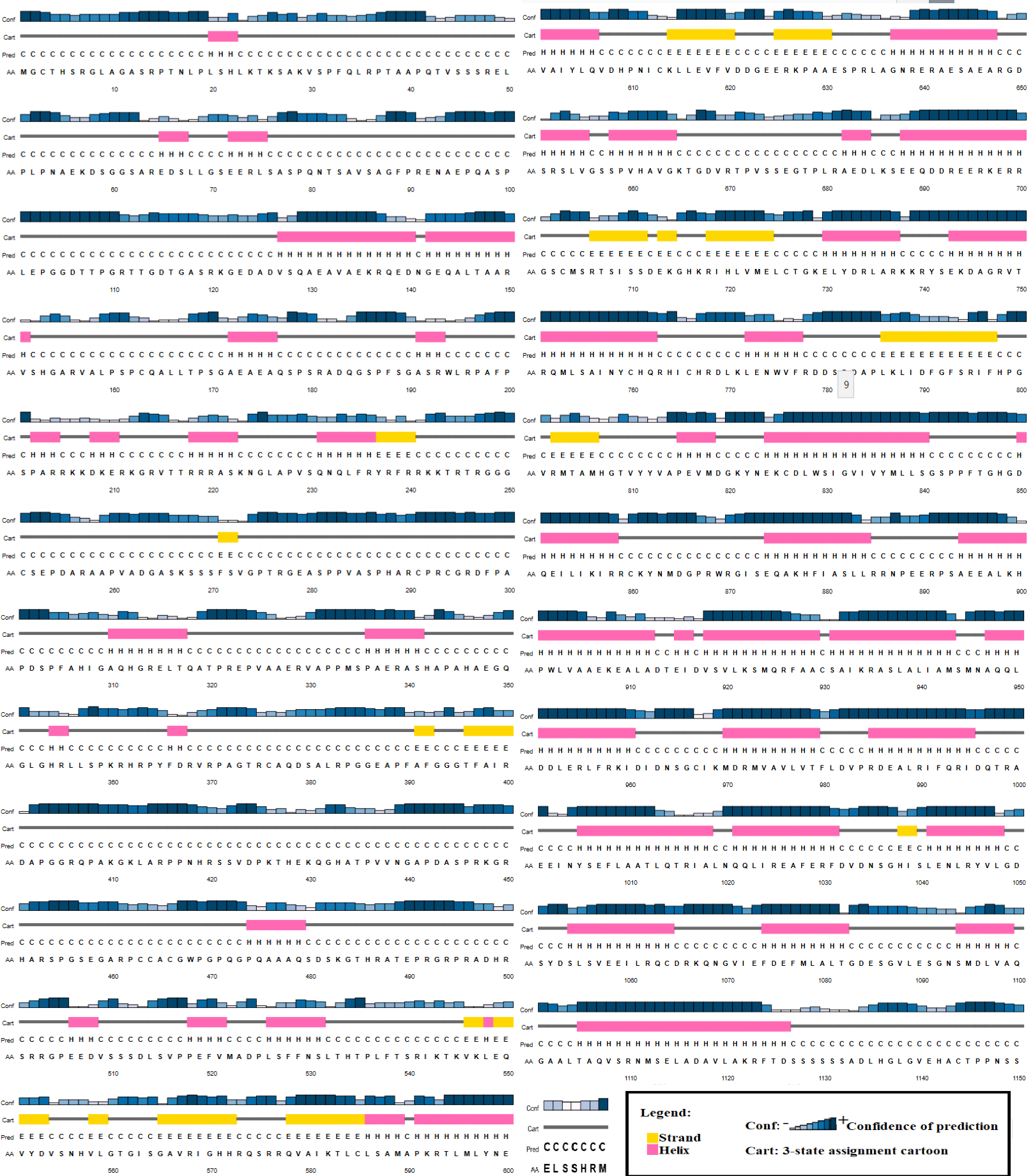


**Additional file 2: Figure S4.** Graphical output from prediction of secondary structure of CDPK4 using PSIPRED tool.


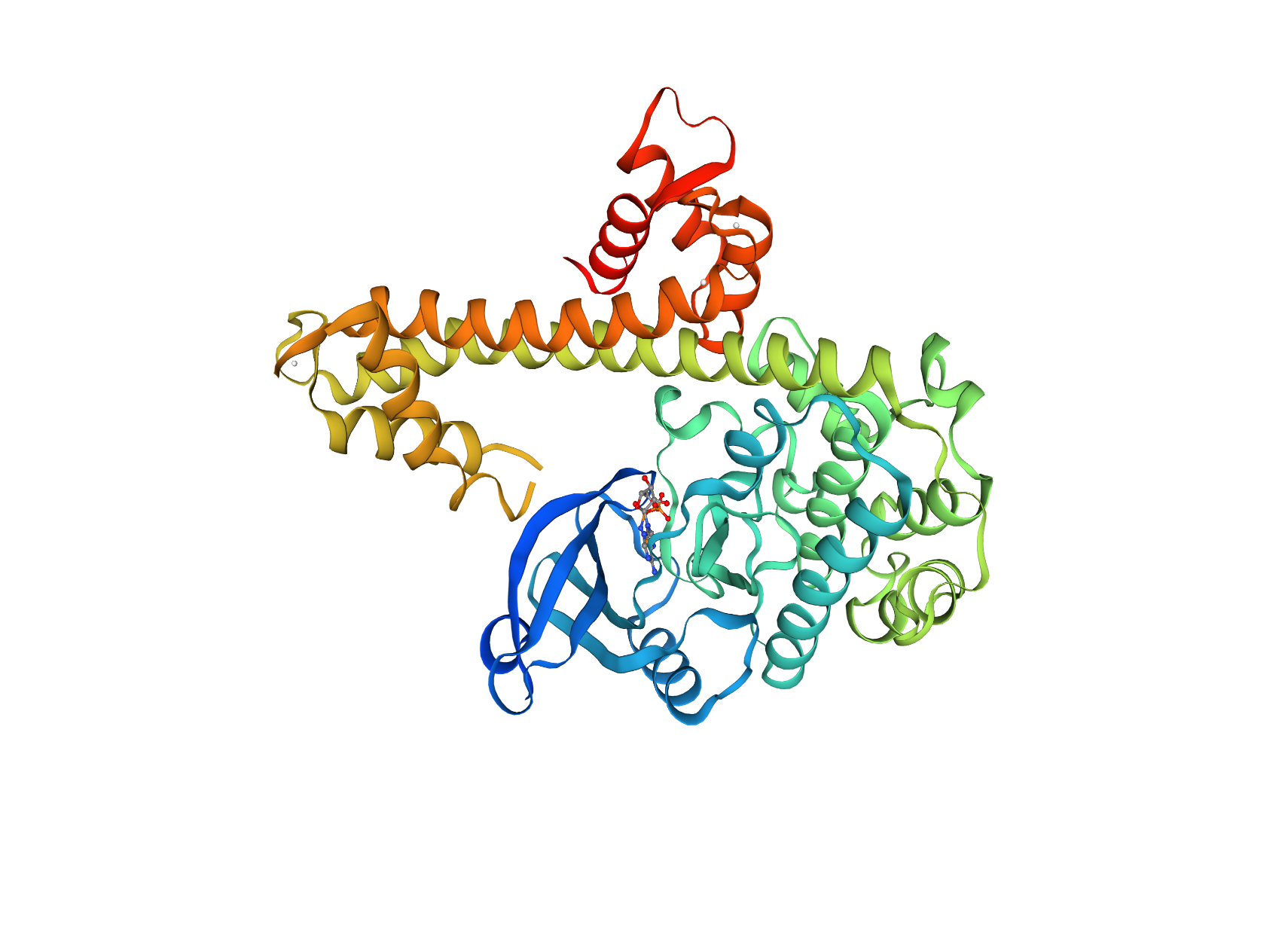


**Additional file 2: Figure S5.** Predicted 3D model by the SWISS-MODEL server.


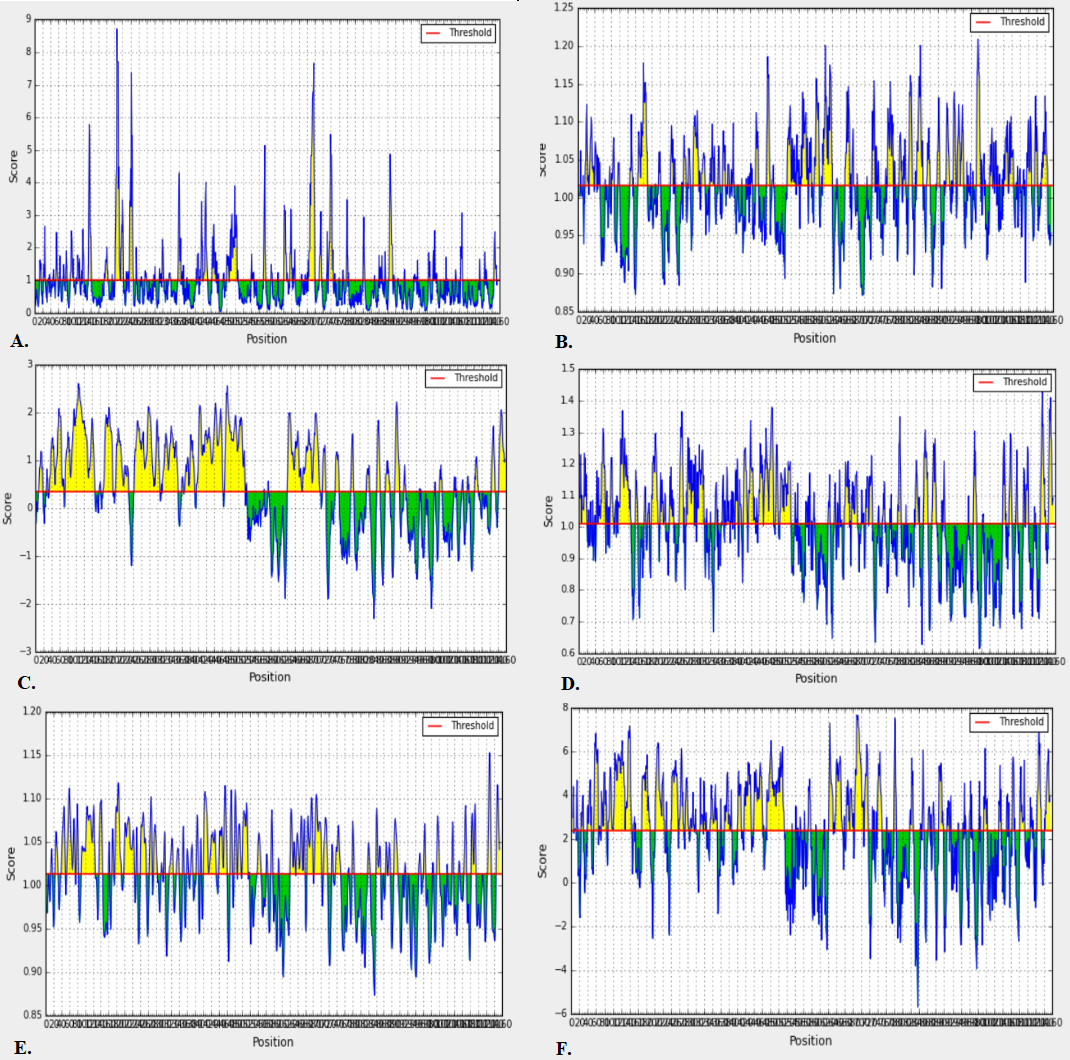


**Additional file 2: Figure S6. Propensity scale plots of CDPK4 protein.**

**(A)** Surface accessibility; **(B)** Antigenicity; **(C)** Bepipred linear epitope prediction; **(D)** Beta-turn; **(E)** Flexibility; **(F)** Hydrophilicity. On the graphs, the Y-axes indicate the corresponding score for each residue (averaged in the specified window), while the X-axes indicate the residue positions in the sequence. The higher residue score could be interpreted as having a higher likelihood that the residue would be part of the epitope (yellow color on the graphs). Green color (under the threshold) shows the unfavorable regions that are related to the properties of interest.
